# The reciprocity of skeletal muscle and bone: an evolving view from mechanical coupling, secretory crosstalk to stem cell exchange

**DOI:** 10.3389/fphys.2024.1349253

**Published:** 2024-03-04

**Authors:** Hao Sui, Jinfeng Dou, Bing Shi, Xu Cheng

**Affiliations:** State Key Laboratory of Oral Diseases and National Clinical Research Center for Oral Diseases, West China School of Stomatology, Sichuan University, Chengdu, China

**Keywords:** musculoskeletal system, inter-tissue communication, muscular diseases, bone regeneration, fibro-adipogenic progenitors

## Abstract

**Introduction:** Muscle and bone constitute the two main parts of the musculoskeletal system and generate an intricately coordinated motion system. The crosstalk between muscle and bone has been under investigation, leading to revolutionary perspectives in recent years.

**Method and results:** In this review, the evolving concept of muscle-bone interaction from mechanical coupling, secretory crosstalk to stem cell exchange was explained in sequence. The theory of mechanical coupling stems from the observation that the development and maintenance of bone mass are largely dependent on muscle-derived mechanical loads, which was later proved by Wolff’s law, Utah paradigm and Mechanostat hypothesis. Then bone and muscle are gradually recognized as endocrine organs, which can secrete various cytokines to modulate the tissue homeostasis and remodeling to each other. The latest view presented muscle-bone interaction in a more direct way: the resident mesenchymal stromal cell in the skeletal muscle, i.e., fibro-adipogenic progenitors (FAPs), could migrate to the bone injury site and contribute to bone regeneration. Emerging evidence even reveals the ectopic source of FAPs from tissue outside the musculoskeletal system, highlighting its dynamic property.

**Conclusion:** FAPs have been established as the critical cell connecting muscle and bone, which provides a new modality to study inter-tissue communication. A comprehensive and integrated perspective of muscle and bone will facilitate in-depth research in the musculoskeletal system and promote novel therapeutic avenues in treating musculoskeletal disorders.

## 1 Introduction

The skeletal muscle and the bone constitute the two major components of the musculoskeletal system. They are in charge of the voluntary body movement, ranging from walking, jumping, facial expression, mastication to respiration. It has long been assumed that muscle and bone are a structural and mechanical integrity. The anatomical adjacency and mechano-transduction inside the tissues facilitate their growth and functional fulfillment. Furthermore, studies in the past 20 years highlighted the endocrine roles of muscle and bone, opening more avenues to the interaction between the two tissues. It was not until recently that a direct connection had been established. The resident mesenchymal stromal cell in the skeletal muscle, i.e., fibro-adipogenic progenitors (FAPs), could migrate to the bone injury site and contribute to bone regeneration. This three-stage view of the muscle and bone relationship has greatly expanded the connotation of the musculoskeletal system and set up a new model for inter-organ communication.

## 2 Mechanical coupling

Because of the principal role in locomotion, the first noticed and studied aspect of muscle-bone interaction was mechanical. Anatomically, bones are “cornerstones” and muscles are more like “appendants”: skeletal muscles connect bones and joints through tendons, while bones provide firm attachment sites in return (Frontera and Ochala, 2015). In contrast, muscles are “commanders” and bones are “executors” when completing physical activities. During physical activities, skeletal muscles are specified and actuated by the nervous system (Jayasinghe et al., 2022), followed by the asynchronous slide of actin and myosin filaments, which induces muscle contraction and thus generates a variety of motion-torque patterns at joints (Sweeney and Hammers, 2018; Ludvig et al., 2022), enabling the multidirectional movement of bones (Sylvester et al., 2021).

The muscle and the bone also interact through mechanical loads that culminate in remodeling (Goodman et al., 2015). The mechanical load exerted by bones on muscles originates from the elastic force accumulated by the deformation of bones under stress and has not been accurately measured. But mechanical loads imposed by muscles on bones are easier to comprehend and appreciate. During embryogenesis, muscle serves as a functional force generator early in development, exerting an increased mechanical load that can be translated into signals that combine with the genetic program of organogenesis on neighboring tissues as development proceeds (Felsenthal and Zelzer, 2017) and lasts throughout life. Most importantly, the development and maintenance of bone mass are largely dependent on muscle-derived mechanical loads (Goodman et al., 2015). As Wolff’s law pointed out, bone size and geometry would change according to the strain applied. It was calculated that force produced in muscle contraction accounted for more than 70% of the bending moments imposed on the lower limb. Therefore, skeletal muscle was considered the primary source of mechanical loading of the bone, which became the central principle of the Utah paradigm. It was thus reasonable to infer that bone mass and mechanical properties would be allometric scaled to the peak muscle force. To depict a comprehensive prospect of the mechanical coupling, mechanostat hypothesis put forward a disuse-adapted-overload model where force over 3,000 microstrain would cause bone formation, while force under 5,00 microstrain would lead to bone resorption. In addition to generating growth stimulus on the bone, the skeletal muscle can also exacerbate the bone growth defect, as seen in the adolescent idiopathic scoliosis (Fadzan and Bettany-Saltikov, 2017). Strong paravertebral muscle on the convex side will reduce the mechanical loading on the spinal bone, thus promoting the bone growth. While weak paravertebral muscle on the concave side can increase the load and impede bone growth, resulting in aggravated curvature (Shao et al., 2023).

The Wolff’s law, the Utah paradigm and the mechanostat hypothesis lay the foundation for the muscle-bone mechanical coupling theory. As the theory postulated, the growth in muscle strength should precede the growth in bone strength, which was proved by a longitudinal study examining the pubertal growth revealing that the peak of lean body mass preceded the peak of bone mineral content by an average of 0.51 years in girls and 0.36 years in boys (Rauch et al., 2004). Similarly, in a mice hindlimb suspension study, it was demonstrated that the cortical thickness of femur and tibia decreased 7 days later than the loss of gastrocnemius and quadricep muscle mass (Wang et al., 2020). These later obeservations verified the theoretic deduction.

## 3 Secretory crosstalk

Apart from generating and maintaining strength by mechanical coupling, the musculoskeletal system also influences the metabolism and function of other organs, including multi-organ insulin sensitivity (Bowden Davies et al., 2018), cardiac health (Wu et al., 2021) and risk of cancer (Jurdana, 2021). Since 1960s, researchers had hypothesized that skeletal muscle possess “humoral” factors, because electrical stimulation of dysfunctional muscles in patients with spinal cord injury induces many of the same physiological changes as in uninjured individuals (Kjaer et al., 1996; Mohr et al., 1997), implying that the musculoskeletal system can affect multiple organs in a non-neural conduction manner. The identification of myostatin confirms the existence of muscle secretory factor, which functions specifically as a negative regulator of skeletal muscle growth (McPherron et al., 1997). Since then, more than 650 myokines have joined the growing list of muscle secretory factors (Khan and Ghafoor, 2019). These muscle secretory factors that are produced, expressed and released by muscle fibers and exert either autocrine, paracrine or endocrine effects are classified as myokines (Severinsen and Pedersen, 2020), making skeletal muscle more than just a component in our locomotor system. When it comes to the other player in the musculoskeletal system, bone is generally thought as a torpid organ. But in recent years, studies have shown an active role of bone. It can function as an endocrine organ by producing many cytokines and proteins called osteokines to modulate glucose and energy metabolism as well as phosphate metabolism (Wang et al., 2021a).

### 3.1 Muscle secretory factors to bone

**FIGURE 1:**
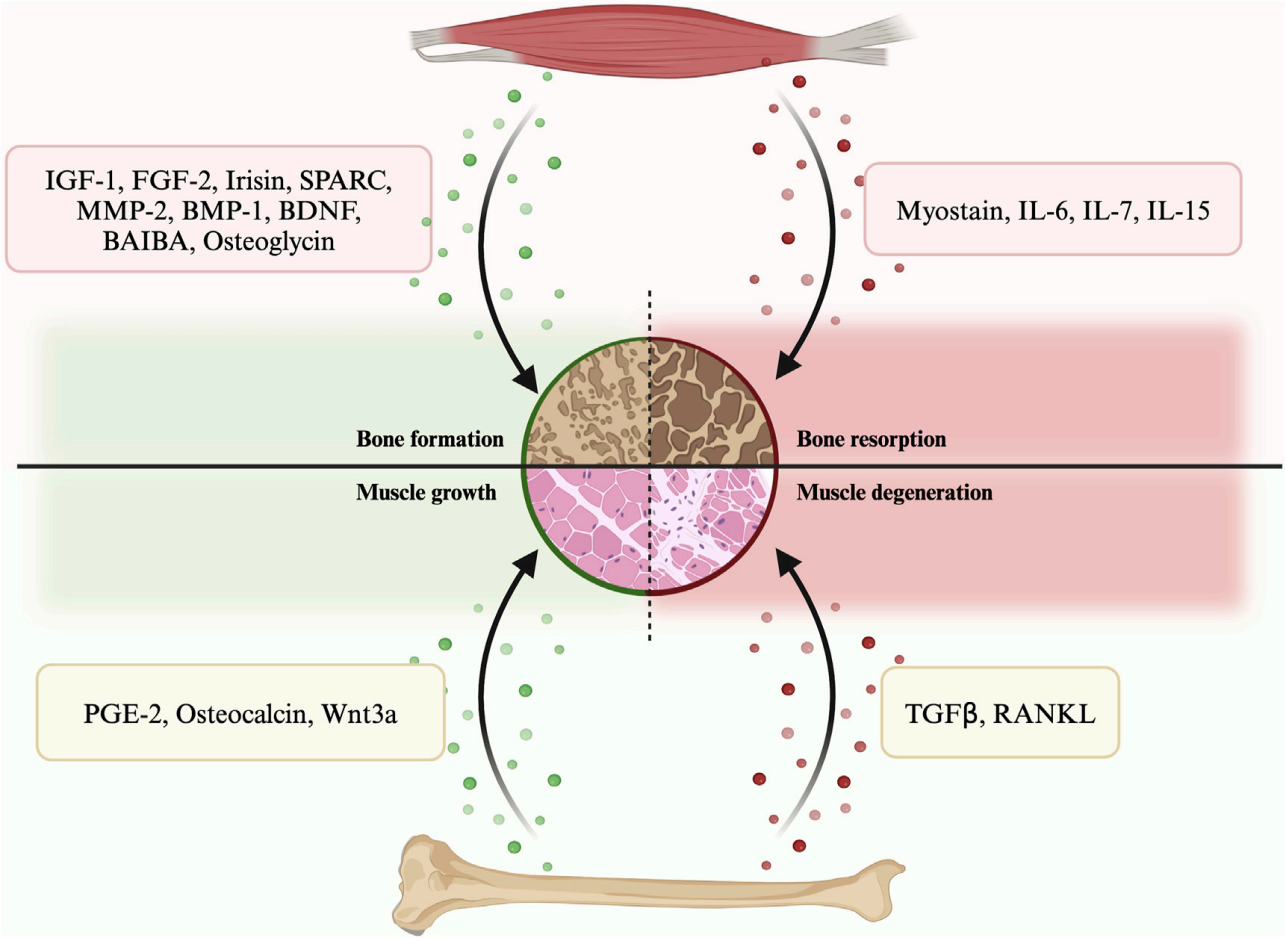
A summary of the secretory crosstalk between muscle and bone.

In terms of their effects on bone regeneration, myokines can be categorized into two types: bone formation factors and bone resorption factors (Figure 1; Supplementary Table S1). The former type includes various myokines such as insulin-like growth factor 1 (IGF-1), fibroblast growth factor 2 (FGF-2), irisin, secreted protein acidic and rich in cysteine (SPARC), matrix metalloproteinase 2 (MMP-2), bone morphogenetic protein 1 (BMP-1), brain-derived neurotrophic factor (BDNF), β-aminoisobutyric acid (BAIBA) etc. On the other hand, myokines that promote bone resorption include myostatin (GDF-8), interleukins, etc. These myokines play a crucial role in bone remodeling and regeneration.

#### 3.1.1 Bone formation myokines

The established role of IGF-1 and FGF-2 in bone physiology pertains to their ability to maintain proper bone matrix levels and bone mass by promoting osteoblast proliferation and differentiation (Novais et al., 2021; Chen et al., 2022; Mazziotti et al., 2022). *In vitro* studies demonstrated that myotubes secreted IGF-1 and FGF-2 (Clarke and Feeback, 1996; Henningsen et al., 2010), while *in vivo* experiments revealed high concentrations of IGF-1 in wound secretions from muscle flaps and increased local FGF-2 release following muscle injury (Vogt et al., 2005; D’Amore et al., 1994), in addition to elevated circulating FGF-2 levels (D’Amore et al., 1994). Furthermore, the localization of IGF-1 and FGF-2 at the muscle-periosteum interface suggested that muscle-derived IGF-1 and FGF-2 may play a role in bone regeneration through paracrine or endocrine actions (Hamrick et al., 2010).

Irisin, a hormone induced by exercise, is a fragmented product of fibronectin type III domain-containing protein 5(FNDC-5) and acts as a linkage between muscles and other tissues (Waseem et al., 2022). It has been demonstrated that Irisin expression is positively associated with bone mineral density and bone strength (Singhal et al., 2014; Palermo et al., 2015; Colaianni et al., 2019). More recently, irisin has also been shown to have anabolic effects on bone in animal models. Increased circulating irisin levels enhance osteogenesis in mice by inducing osteoblastogenesis and inhibiting osteoclastogenesis in bone cell lines (Zhang et al., 2017a). And the administration of irisin prevents both disuse-induced and peroxide–induced osteocyte apoptosis (Kim et al., 2018; Storlino et al., 2020). On the other hand, Irisin acts directly on osteoclast progenitors to increase differentiation and promote bone resorption, implying it may also serve as an important counter-regulatory hormone that targets both osteoclasts and osteocytes (Estell et al., 2020).

SPARC is a glycoprotein that plays a crucial role in various physiological processes such as extracellular matrix remodeling, tissue repair, and collagen mineralization promotion (Zhou et al., 2018). During muscle damage and regeneration, SPARC is notably upregulated (Jørgensen et al., 2009). The SPARC null mice was characterized by delayed fibrocartilage mineralization that leads to the loss of bone mass (Wang et al., 2021b), suggesting that SPARC positively regulates bone regeneration and facilitates fibrocartilage mineralization, thereby aiding bone regeneration.

Matrix metalloproteinase 2 (MMP-2), a member of the extracellular matrix metalloproteinase family, is actively secreted by myotubes *in vitro*. The protein is essential for maintaining bone mineral density and strength, as well as skeletal development (Hardy and Fernandez-Patron, 2020). In MMP-2 null mice, bone loss and reduced bone mineral density occurred, meanwhile the lack of MMP-2 affected the later stages of fracture healing tissue remodeling (Lieu et al., 2011). However, dysregulated MMP-2 expression also led to various diseases, including developmental osteolysis and metastatic osteolysis (Li et al., 2021). Although MMP-2 may have a potential role in promoting bone formation as a myokine, further exploration is necessary to understand its specific function. Bone morphogenetic protein 1 (BMP-1), a zinc-dependent metalloproteinase, promoted bone mineralization (Kessler et al., 1996; Kudo, 2019). Expression of BMP-1 was detected in human myotubes cultured *in vitro* and in muscle tissues subjected to blast trauma (Hittel et al., 2009; Jackson et al., 2011), which could partially explain the ectopic ossification after blast trauma (Jackson et al., 2011). However, the role of BMP-1 as a myokine in normal bone regeneration requires further investigation.

Brain-derived neurotrophic factor (BDNF) is a member of the neurotrophin family of growth factors (Colucci-D’Amato et al., 2020). In addition to its role in neuron growth, neuronal development, and synaptic plasticity and function, BDNF is also expressed and released by skeletal muscle as a myokine that is capable of enhancing lipid oxidation in muscles via activation of AMPK (Pedersen et al., 2009; Antony and Li, 2020). Physical activities has a positive effect on the release of BDNF (Kim and Kim, 2018; Behrendt et al., 2021), whose specific receptor tropomyosin-related kinase B (TrkB) was shown to be expressed at high levels in the osteoblasts (Asaumi et al., 2000). During fracture healing, BDNF was found to positively modulate the expression and secretion of VEGF from osteoblasts via TrkB/ERK1/2 signaling pathway (Zhang et al., 2017b). And AKT signaling was found to be activated downstream, followed by the upregulation of integrin β1, therefore stimulating osteoblasts migration (Zhang et al., 2020). A recent study reported BDNF was related to the maintenance of mitochondrial quality (Ahuja et al., 2022). Taking it into consideration that mitochondrial dysfunction is involved in several degenerative bone and joint diseases, especially osteoporosis and osteoarthritis (Lee et al., 2021; Sun et al., 2021), BDNF may play a more important role in bone remodeling than it does in our current knowledge.

β-aminoisobutyric acid (BAIBA) origins from mitochondrial valine catabolism and is increased by physical activities (Kamei et al., 2020). BAIBA served as a bone-protective factor that prevents osteocyte cell death induced by reactive oxygen species (Kitase et al., 2018). Osteoglycin is a secreted protein found in skeletal muscle production and is recognized as a crucial anabolic factor produced by muscle-derived cells. Its secretion into the bloodstream promoted bone formation (Tanaka et al., 2012).

#### 3.1.2 Bone resorption myokines

Myostatin (GDF-8), known as a retro-myokine, is secreted by muscle and acts as an inhibitor of muscle hypertrophy (McPherron et al., 1997; Kim et al., 2021). It is expressed in fracture healing tissue and suppresses the initial recruitment and proliferation of osteogenic progenitor cells in the healing tissue (Kellum et al., 2009). Myostatin could impede osteoblast differentiation and activate osteoclast maturation, resulting in compromised bone structure, bone density, and contractile properties (Bialek et al., 2014; Suh et al., 2020; Zhi et al., 2020; Liu et al., 2021; Omosule et al., 2022; Tang et al., 2022).

Interleukin (IL) families are pro-inflammatory mediators secreted by various cell types across the body. Several ILs including IL-6, IL-7, IL-15 were identified as myokines. During physical activities, circulating IL-6 level surged, and the majority of which was proven to originate from muscle (Chowdhury et al., 2020). IL-6 drived osteoclastogenesis and led to a bone-resorbing outcome (Udagawa et al., 1995; Chowdhury et al., 2020). IL-7 and IL-15 are also secreted by skeletal muscle and could induce bone resorption either directly by inducing osteoclastogenesis (Ogata et al., 1999; Kim et al., 2017), or indirectly by acting on effector cells consisting macrophages and NK cells (Feng et al., 2015; Kim et al., 2020). However, there is also an opposite perspective for IL-15, suggesting it is crucial for osteoblastic matrix formation and bone mineralization (Loro et al., 2017).

All myokines mentioned above are involved in regulating bone metabolism through endocrine pathways, which ultimately resulted in promotion or inhibition of bone remodeling.

### 3.2 Bone secretory factors to muscle

According to their impacts on muscle, osteokines can be categorized into muscle growth osteokines and muscle degeneration osteokines. Examples of the former include prostaglandin E2 (PGE-2), osteocalcin and Wnt3a, while transforming growth factor β (TGFβ) and receptor activator of nuclear factor kappa-Β ligand (RANKL) belong to the latter. Since the concept of bone as an endocrine organ is relatively new, studies on osteokines are less in-depth than those on myokines.

#### 3.2.1 Muscle growth oseokines

The osteokine PGE-2 is secreted at high levels by osteocytes, which has four types of receptors executing different functional roles. Stimulating the receptor EP4 significantly enhanced myoblast proliferation (Mo et al., 2015). While down-regulating the receptor EP2 promoted the fusion of human muscle progenitors *in vitro* and improved their transplantation capability (Sakai-Takemura et al., 2020). Osteocalcin is mainly produced by mature osteoblasts, but also by osteocytes (Bonewald, 2019). And pervious study has shown osteocalcin favored uptake and catabolism of nutrients in muscle and was necessary for adaptation to exercise (Mera et al., 2016). Wnt signaling is involved in the control of the myogenic program and the differentiation of satellite cells, the muscle resident stem cells (Fujimaki et al., 2014). A couple of Wnt signaling pathway-related factors were reported to support myogenesis and muscle function. Wnt1 is highly expressed in osteocytes and Wnt3a is produced by osteocytes in response to shear stress. They could both induce myogenesis but Wnt3a seems to be more potent than Wnt1 (Huang et al., 2017).

#### 3.2.2 Muscle degeneration osteokines

TGFβ is mainly produced by bone-forming osteoblasts (Bonewald, 2019). In osteolytic bone metastases cancer models, TGFβ were released from the bone surface, and resulting in elevated oxidization of skeletal muscle proteins that contributed to muscle weakness (Waning et al., 2015). RANKL was first identified as a product of immune cells, but has since been shown to be produced by osteocytes to activate osteoclasts (Xiong et al., 2011). Injections of RANKL inhibitor to mice significantly increased the force of dystrophic EDL muscle (Dufresne et al., 2018), suggesting RANKL is a promising target to control muscle remodeling.

## 4 Stem cell exchange

As the researches delve deeper, it came out that muscle-bone interaction went beyond mechanical and paracrine: stem cells from the skeletal muscle could actually migrate to the underlying bone and directly contribute to bone regeneration. On the other hand, there is currently no evidence of stem cell contribution from the bone to the skeletal muscle, which prompts us to view muscle as a more vigorous part in this pair.

### 4.1 Stem cell contribution from muscle to bone: traditional view and new perspective of skeletal stem cells

Bone maintains its structural integrity and functionality through a process that relies heavily on the activation of skeletal stem/progenitor cells (SSPCs) (Jeffery et al., 2022). These specialized cells are capable of both self-renewal and multilineage differentiation into bone, cartilage, and stroma (Chan et al., 2018). Multiple sources of skeletal stem/progenitor cells (SSPCs) have been identified for bone repair, including bone marrow, growth plate, periosteum. Each population displays unique lineage capabilities and is involved in bone repair in varying degrees (Serowoky et al., 2020). SSPCs derived from bone marrow are primarily identified by the Leptin receptor (LepR) and are responsible for generating new osteoblasts in adult bone marrow, which can form ossicles supporting hematopoiesis *in vivo* (Zhou et al., 2014; Matsushita et al., 2020). Growth plate SSPCs express parathyroid hormone-related protein (PTHrP) and maintain the resting zone while providing a source of chondrocytes during bone repair (Mizuhashi et al., 2018). In the periosteum, Gil1+ SSPCs contribute to and are required for the growth and repair of skull bones (Zhao et al., 2015), whereas Prrx1+ cells broadly mark periosteal SSPCs in the limbs and elsewhere (Duchamp de Lageneste et al., 2018), re-establishing the pool of bone progenitor cells after injury. Additionally, Ctsk is a conservative marker of periosteal SSPCs, with Ctsk + cells contributing primarily to osteoblasts in cortical bone (Debnath et al., 2018).

In reconstructive surgeries, fractured bone with a muscular flap always heals better. Consequently, it has long been assumed that the skeletal muscle tissue plays a protective role in bone fracture repair. But little was known about the cellular and molecular process. The Colnot team endeavored to explore the involvement of muscle-originated stem cells in bone fracture healing (Abou-Khalil et al., 2015). At first, they investigated the contribution of muscle satellite cells (MuSCs), with lineage-tracing transgenic mice and transplantation experiments. However, they found rare MuSCs could form chondrocyte and the MuSCs contribution in the bone fracture callus was not substantial. In the end, they identified the critical role of BMP-2, which was secreted by MuSCs and promoted bone fracture healing. This was the first evidence that cells derived from muscle contributed to bone regeneration, though in a paracrine way. For further study, the Colnot team investigated another stem cell resident in the skeletal muscle—fibro-adipogenic progenitors (FAPs), which are the mesenchymal stem cells closely coordinated with MuSCs in the orchestration of skeletal muscle regeneration. The authors transplanted EDL muscle grafts from Prrx1Cre; RosamTmG mice donors into wild-type hosts. They observed that progenitors originated from skeletal muscle gave rise to chondrocytes and osteoblasts for bone repair. Further scRNAseq analyses identified these progenitors as FAPs within skeletal muscle but expressing common markers with SSPCs. They also built a polytrauma mouse model to investigate the role of FAPs in musculoskeletal trauma and found that FAPs failed to undergo chondrogenesis under polytrauma, resulting in the non-union phenotype of fracture. Their sophisticated work verified the direct contribution of FAPs to bone repair and highlight the role of injury in mediating FAPs’ behavior. FAPs has thus been confirmed as a new source of SSPCs.

### 4.2 Stem cell contribution from bone to muscle: an unanswered question

Although the myogenic capacity of SSPCs has not been extensively studied, the simultaneous occurrence of bone fragility and muscle weakness in osteogenesis imperfecta (OI) patients suggests a potential link between these two tissues (Phillips and Jeong, 2018). It has been reported that the mechanotransduction and functionality of the muscle-bone unit was repaired in OI (Veilleux et al., 2015). Whether there could be defects in the direct intercellular communication between bone and muscle stem cells still warrants further investigation. Exploring the underlying mechanisms responsible for the decline of both bone and muscle could lead to the development of novel physiotherapeutic and pharmacological interventions for OI and other musculoskeletal disorders.

## 5 Discussion

### 5.1 FAPs–the critical cell connecting muscle and bone

Ever since its verified role as an essential progenitor cell in skeletal muscle in 2010, the FAP cell has been under energetic investigation. And the discovery that FAPs can also serve as SSPCs enhanced our comprehension of the musculoskeletal system. Evidence from studies of FAPs in skeletal muscle homeostasis maintenance and regeneration may provide inspiration for the roles of FAPs and other SSPCs in the bone, and *vice versa*.

FAPs are quiescent mesenchymal stromal cells with multipotency to differentiate into all the mesenchymal lineages, depending on the context of tissue damage (Contreras et al., 2021). The systemic protease, hepatocyte growth factor activator, which was induced by tissue injury, could prime FAPs to transitions from quiescence to G alert state (Rodgers et al., 2017). TGFβ signaling remained the most studied signaling pathway regulating FAPs’ fate and behavior. Ligands of the TGFβ super family, including TGFβ, myostatin and bone morphogenetic proteins (BMPs) could induce cell proliferation, myofibroblast differentiation and extracellular matrix (ECM) deposition (Juban et al., 2018). Wnt/β-catenin signaling and platelet-derived growth factor signaling could also activate FAPs and induce the expression of several ECM genes (Akhmetshina et al., 2012; Contreras et al., 2019). On the other hand, tumor necrosis factor-alpha (TNFα) induced the apoptosis of FAPs (Lemos et al., 2015). As for the adipogenic differentiation of FAPs, the cellular communication network (CCN) family members and dexamethasone could play a stimulative role, while IL4 and histone deacetylase inhibitors could play a suppressive role (Mozzetta et al., 2013; Dong et al., 2014; Hu et al., 2019). In addition, TGFβ signaling can inhibit the adipogenic differentiation of FAPs (Contreras et al., 2021). It has also been reported that FAPs were the main cell responsible for intramuscular ossification. Both BMP2 and BMP9 could promote FAP osteogenic differentiation (Shore, 2011). Moreover, targeted expression of an activin receptor in FAPs could recapitulate full spectrum of heterotopic ossification in muscle, suggesting the key role of activin signaling in regulating FAP osteogenic differentiation (Lees-Shepard et al., 2018). When applied at a low concentration, TNFα could promote FAP osteogenic differentiation in the context of bone fracture healing (Glass et al., 2011).

Recent studies revealed that FAPs have different embryonic origins, similar to the muscle they reside in (Sefton and Kardon, 2019). While limb muscles arise from somites, craniofacial muscles originate from branchial arches. Craniofacial muscles exhibit delayed myofiber reconstitution and prolonged fibrosis during repair, in contrast to somite-derived limb muscles, where FAPs serve as the key mediator of muscle fibrosis (Cheng et al., 2021; Cheng et al., 2022). It has been demonstrated in studies of skin and mucosa that cells derived from neural crest and mesoderm have distinct fibrogenic potential (Rinkevich et al., 2015; Pratsinis et al., 2019). Thus the branchiomeric FAPs in the craniofacial muscle could be the main culprit for the impaired muscle regeneration.

It is also observed in the bone that cells from different origins demonstrated distinct behaviors. But the scenario is more complicated. When the bone is injured, the neural crest-derived mandible fully regenerated with neural crest-derived SSPCs, and the mesoderm-derived tibia heals with mesoderm-derived SSPCs (Leucht et al., 2008). Further transplantation experiment revealed that the SSPCs from different lineages were functionally interchangeable only when the host and the donor had the same hox code (Leucht et al., 2008). And this offers another possible explanation for the muscle: the mesoderm-derived myofiber and neural crest-derived FAPs are mutually exclusive, leading to impaired regeneration process. However, there is currently no research exploring the osteogenic potential of FAPs from different embryonic origins, neither *in vitro* nor in the scenario of bone regeneration. It is interesting to figure out whether the beneficial role of FAPs in bone regeneration is dependent on their embryonic origin. It is also of great clinical importance to guide the flap transfer surgery.

### 5.2 Therapeutic possibilities involving FAPs for treating musculoskeletal diseases

The perspective of FAPs as an active stem cell participant in neighboring tissue regeneration offers more possibilities for regenerative medicine and orthobiologics for musculoskeletal disorders. Platelet-rich plasma (PRP), bone marrow and adipose tissue are the most commonly used orthobiologics (Fang and Vangsness, 2021). PRP and bone marrow aspirations have the advantage of being minimally manipulated but have a relatively low concentration of stem cell components, which can largely aid tissue regeneration (Fang and Vangsness, 2021). Since mesenchymal stem cells (MSCs) are the most prospective stem cell for regenerative medicine (Lemos and Duffield, 2018), adipose tissue and umbilical cord, which are rich in MSCs, are believed to be promising. Adipose tissue can yield the highest number of MSCs per milliliter of tissue while Whartons jelly tissue can provide the highest concentration of MSCs (Shapiro et al., 2022). Nevertheless, the use of adipose and umbilical cord tissue for treating musculoskeletal diseases is either off-shelf or still under investigation (Shapiro et al., 2023). The proportion of FAPs in skeletal muscle tissue is comparable to that of MSCs in the adipose tissue (Giordani et al., 2019), making them another possible choice for stem cell therapies, though the efficiency and security issues are to be scrutinized. Meanwhile, the secretory factors of FAPs also stand a chance of turning into potential therapeutics for promoting bone and muscle regeneration and warrants further investigation. Furthermore, since several immunomodulation agents hold greate promise in several preclinical studies to treat muscle and bone injuries, the immunomodulatory role of FAPs might also facilitate musculoskeletal tissue regeneration (Maruyama et al., 2020; Luo et al., 2022; Duda et al., 2023; Qin et al., 2023).

Potential therapeutic avenues involving FAPs for musculoskeletal disorders have been proposed by a few studies. Nilotinib, a clinically approved tyrosine kinase inhibitor, could exert anti-fibrotic effects on skeletal muscle by restoring FAP apoptosis (Lemos et al., 2015). Another member of the tyrosine kinase inhibitor family, Imatinib, could improve bone regeneration by decreasing the persistent callus fibrosis, which was mainly caused by FAP, in the context of bone-muscle polytrauma (Julien et al., 2021). In addition, the observation that the endothelin receptor type B (EDNRB) was highly expressed in fibrotic FAPs uncovered the critical role of endothelin in the altered crosstalk between muscle cells and FAPs (Bensalah et al., 2022). The application of Bosentan, an antagonist against EDNR could counteract fibrosis and enhance skeletal muscle regeneration (Bensalah et al., 2022).

**FIGURE 2:**
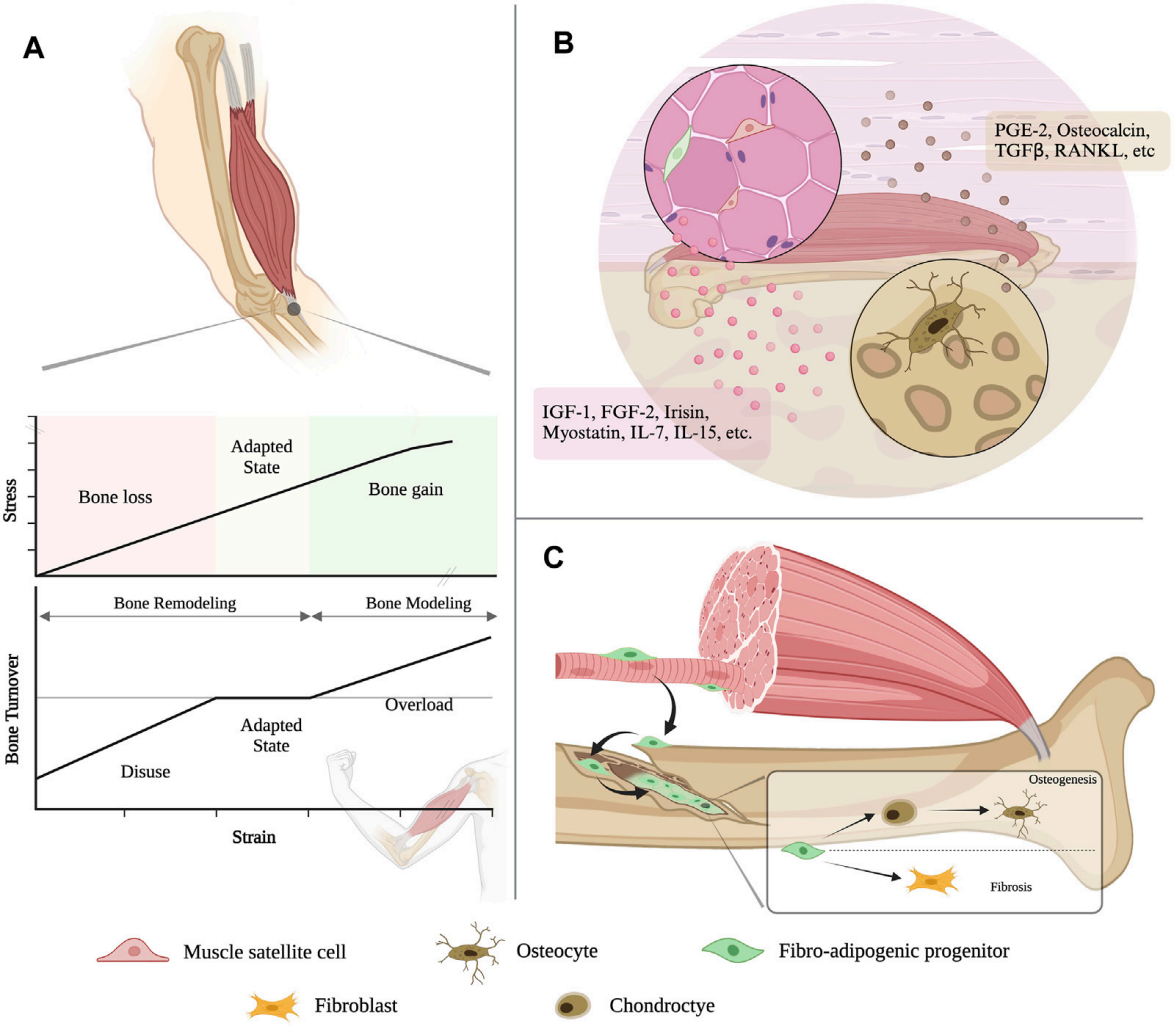
An evolving view of bone-muscle interaction. **(A)** Muscle contraction generates a variety of motion-torque patterns at bone and enables the multidirectional movement of the musculoskeletal system. **(B)** Muscle and bone can both function as endocrine organs, which can secrete various cytokines to modulate the tissue homeostasis and remodeling to each other. **(C)** The resident mesenchymal stromal cell in the skeletal muscle, i.e., fibro-adipogenic progenitors (FAPs), could migrate to the bone injury site and contribute to bone regeneration.

The view of the muscle-bone dialogue has evolved far from mechanical coupling and secretory crosstalk (Figure 2). After FAPs had been identified as the substantial cell contributing to bone fracture healing, cellular exchanges between another two juxtaposed tissues started to be uncovered. Subcutaneous adipose tissue can provide regenerative cells responsible for skeletal muscle regeneration (Sastourné-Arrey et al., 2023). This not only offers more solid evidence to support the existing perspective that bone, muscle and fat work synergistically as a functional unit, but also renders a modality for studying inter-tissue communication in general and brings revelation for future investigations into the cellular crosstalk in multi-organ syndromes, such as cancer cachexia.

## References

[B1] Abou-Khalil R. Yang F. Lieu S. Julien A. Perry J. Pereira C. (2015). Role of muscle stem cells during skeletal regeneration. Stem Cells 33, 1501–1511. 10.1002/stem.1945 25594525

[B2] Ahuja P. Ng C. F. Pang B. P. S. Chan W. S. Tse M. C. L. Bi X. (2022). Muscle-generated BDNF (brain derived neurotrophic factor) maintains mitochondrial quality control in female mice. Autophagy 18, 1367–1384. 10.1080/15548627.2021.1985257 34689722 PMC9225428

[B3] Akhmetshina A. Palumbo K. Dees C. Bergmann C. Venalis P. Zerr P. (2012). Activation of canonical Wnt signalling is required for TGF-β-mediated fibrosis. Nat. Commun. 3, 735. 10.1038/ncomms1734 22415826 PMC3316881

[B4] Antony R. Li Y. (2020). BDNF secretion from C2C12 cells is enhanced by methionine restriction. Biochem. Biophys. Res. Commun. 533, 1347–1351. 10.1016/j.bbrc.2020.10.017 33069357 PMC7744331

[B5] Asaumi K. Nakanishi T. Asahara H. Inoue H. Takigawa M. (2000). Expression of neurotrophins and their receptors (TRK) during fracture healing. Bone 26, 625–633. 10.1016/s8756-3282(00)00281-7 10831935

[B6] Behrendt T. Kirschnick F. Kroger L. Beileke P. Rezepin M. Brigadski T. (2021). Comparison of the effects of open vs. closed skill exercise on the acute and chronic BDNF, IGF-1 and IL-6 response in older healthy adults BMC Neurosci. 22, 71. 10.1186/s12868-021-00675-8 34823469 PMC8614060

[B7] Bensalah M. Muraine L. Boulinguiez A. Giordani L. Albert V. Ythier V. (2022). A negative feedback loop between fibroadipogenic progenitors and muscle fibres involving endothelin promotes human muscle fibrosis. J. Cachexia Sarcopenia Muscle 13, 1771–1784. 10.1002/jcsm.12974 35319169 PMC9178170

[B8] Bialek P. Parkington J. Li X. Gavin D. Wallace C. Zhang J. (2014). A myostatin and activin decoy receptor enhances bone formation in mice. Bone 60, 162–171. 10.1016/j.bone.2013.12.002 24333131

[B9] Bonewald L. (2019). Use it or lose it to age: a review of bone and muscle communication. Bone 120, 212–218. 10.1016/j.bone.2018.11.002 30408611 PMC6360108

[B10] Bowden Davies K. A. Sprung V. S. Norman J. A. Thompson A. Mitchell K. L. Halford J. C. G. (2018). Short-term decreased physical activity with increased sedentary behaviour causes metabolic derangements and altered body composition: effects in individuals with and without a first-degree relative with type 2 diabetes. Diabetologia 61, 1282–1294. 10.1007/s00125-018-4603-5 29671031

[B11] Chan C. K. F. Gulati G. S. Sinha R. Tompkins J. V. Lopez M. Carter A. C. (2018). Identification of the human skeletal stem cell. Cell 175, 43–56. 10.1016/j.cell.2018.07.029 30241615 PMC6400492

[B12] Cheng X. Huang Y. Liu Y. Dou J. Zhao N. Li J. (2022). Head muscle fibro-adipogenic progenitors account for the tilted regeneration towards fibrosis. Biochem. Biophys. Res. Commun. 589, 131–138. 10.1016/j.bbrc.2021.12.009 34915407

[B13] Cheng X. Shi B. Li J. (2021). Distinct embryonic origin and injury response of resident stem cells in craniofacial muscles. Front. physiology 12, 690248. 10.3389/fphys.2021.690248 PMC8281086 34276411

[B14] Chen J. Zhou R. Feng Y. Cheng L. (2022). Molecular mechanisms of exercise contributing to tissue regeneration. Signal Transduct. Target Ther. 7, 383. 10.1038/s41392-022-01233-2 36446784 PMC9709153

[B15] Chowdhury S. Schulz L. Palmisano B. Singh P. Berger J. M. Yadav V. K. (2020). Muscle-derived interleukin 6 increases exercise capacity by signaling in osteoblasts. J. Clin. Invest. 130, 2888–2902. 10.1172/JCI133572 32078586 PMC7260002

[B16] Clarke M. S. Feeback D. L. (1996). Mechanical load induces sarcoplasmic wounding and FGF release in differentiated human skeletal muscle cultures. Faseb J. 10, 502–509. 10.1096/fasebj.10.4.8647349 8647349

[B17] Colaianni G. Faienza M. F. Sanesi L. Brunetti G. Pignataro P. Lippo L. (2019). Irisin serum levels are positively correlated with bone mineral status in a population of healthy children. Pediatr. Res. 85, 484–488. 10.1038/s41390-019-0278-y 30683930

[B18] Colucci-D'Amato L. Speranza L. Volpicelli F. (2020). Neurotrophic factor BDNF, physiological functions and therapeutic potential in depression, neurodegeneration and brain cancer. Int. J. Mol. Sci. 21, 7777. 10.3390/ijms21207777 33096634 PMC7589016

[B19] Contreras O. Cruz-Soca M. Theret M. Soliman H. Tung L. W. Groppa E. (2019). Cross-talk between TGF-β and PDGFRα signaling pathways regulates the fate of stromal fibro-adipogenic progenitors. J. Cell Sci. 132, jcs232157. 10.1242/jcs.232157 31434718

[B20] Contreras O. Rossi F. M. V. Theret M. (2021). Origins, potency, and heterogeneity of skeletal muscle fibro-adipogenic progenitors-time for new definitions. Skelet. Muscle 11, 16. 10.1186/s13395-021-00265-6 34210364 PMC8247239

[B21] D'Amore P. A. Brown R. H. JR. Ku P. T. Hoffman E. P. Watanabe H. Arahata K. (1994). Elevated basic fibroblast growth factor in the serum of patients with Duchenne muscular dystrophy. Ann. Neurol. 35, 362–365. 10.1002/ana.410350320 8122890

[B22] Debnath S. Yallowitz A. R. Mccormick J. Lalani S. Zhang T. Xu R. (2018). Discovery of a periosteal stem cell mediating intramembranous bone formation. Nature 562, 133–139. 10.1038/s41586-018-0554-8 30250253 PMC6193396

[B23] Dong Y. Silva K. A. Dong Y. Zhang L. (2014). Glucocorticoids increase adipocytes in muscle by affecting IL-4 regulated FAP activity. FASEB J. 28, 4123–4132. 10.1096/fj.14-254011 24948596 PMC4139907

[B24] Duchamp De Lageneste O. Julien A. Abou-Khalil R. Frangi G. Carvalho C. Cagnard N. (2018). Periosteum contains skeletal stem cells with high bone regenerative potential controlled by Periostin. Nat. Commun. 9, 773. 10.1038/s41467-018-03124-z 29472541 PMC5823889

[B25] Duda G. N. Geissler S. Checa S. Tsitsilonis S. Petersen A. Schmidt-Bleek K. (2023). The decisive early phase of bone regeneration. Nat. Rev. Rheumatol. 19, 78–95. 10.1038/s41584-022-00887-0 36624263

[B26] Dufresne S. S. Boulanger-Piette A. Bossé S. Argaw A. Hamoudi D. Marcadet L. (2018). Genetic deletion of muscle RANK or selective inhibition of RANKL is not as effective as full-length OPG-fc in mitigating muscular dystrophy. Acta Neuropathol. Commun. 6, 31. 10.1186/s40478-018-0533-1 29699580 PMC5922009

[B27] Estell E. G. Le P. T. Vegting Y. Kim H. Wrann C. Bouxsein M. L. (2020). Irisin directly stimulates osteoclastogenesis and bone resorption *in vitro* and *in vivo* . Elife 9, e58172. 10.7554/eLife.58172 32780016 PMC7444909

[B28] Fadzan M. Bettany-Saltikov J. (2017). Etiological theories of adolescent idiopathic scoliosis: past and present. Open Orthop. J. 11, 1466–1489. 10.2174/1874325001711011466 29399224 PMC5759107

[B29] Fang W. H. Vangsness C. T. JR. (2021). Food and drug administration's position on commonly injected biologic materials in orthopaedic surgery. Am. J. Sports Med. 49, 3414–3421. 10.1177/0363546521990900 33769895

[B30] Felsenthal N. Zelzer E. (2017). Mechanical regulation of musculoskeletal system development. Development 144, 4271–4283. 10.1242/dev.151266 29183940 PMC6514418

[B31] Feng S. Madsen S. H. Viller N. N. Neutzsky-Wulff A. V. Geisler C. Karlsson L. (2015). Interleukin-15-activated natural killer cells kill autologous osteoclasts via LFA-1, DNAM-1 and TRAIL, and inhibit osteoclast-mediated bone erosion *in vitro* . Immunology 145, 367–379. 10.1111/imm.12449 25684021 PMC4479536

[B32] Frontera W. R. Ochala J. (2015). Skeletal muscle: a brief review of structure and function. Calcif. Tissue Int. 96, 183–195. 10.1007/s00223-014-9915-y 25294644

[B33] Fujimaki S. Hidaka R. Asashima M. Takemasa T. Kuwabara T. (2014). Wnt protein-mediated satellite cell conversion in adult and aged mice following voluntary wheel running. J. Biol. Chem. 289, 7399–7412. 10.1074/jbc.M113.539247 24482229 PMC3953255

[B34] Giordani L. He G. J. Negroni E. Sakai H. Law J. Y. C. Siu M. M. (2019). High-dimensional single-cell cartography reveals novel skeletal muscle-resident cell populations. Mol. Cell 74, 609–621. 10.1016/j.molcel.2019.02.026 30922843

[B35] Glass G. E. Chan J. K. Freidin A. Feldmann M. Horwood N. J. Nanchahal J. (2011). TNF-alpha promotes fracture repair by augmenting the recruitment and differentiation of muscle-derived stromal cells. Proc. Natl. Acad. Sci. U. S. A. 108, 1585–1590. 10.1073/pnas.1018501108 21209334 PMC3029750

[B36] Goodman C. A. Hornberger T. A. Robling A. G. (2015). Bone and skeletal muscle: key players in mechanotransduction and potential overlapping mechanisms. Bone 80, 24–36. 10.1016/j.bone.2015.04.014 26453495 PMC4600534

[B37] Hamrick M. W. Mcneil P. L. Patterson S. L. (2010). Role of muscle-derived growth factors in bone formation. J. Musculoskelet. Neuronal Interact. 10, 64–70. 20190381 PMC3753580

[B38] Hardy E. Fernandez-Patron C. (2020). Destroy to rebuild: the connection between bone tissue remodeling and matrix metalloproteinases. Front. Physiol. 11, 47. 10.3389/fphys.2020.00047 32116759 PMC7013034

[B39] Henningsen J. Rigbolt K. T. Blagoev B. Pedersen B. K. Kratchmarova I. (2010). Dynamics of the skeletal muscle secretome during myoblast differentiation. Mol. Cell Proteomics 9, 2482–2496. 10.1074/mcp.M110.002113 20631206 PMC2984231

[B40] Hittel D. S. Berggren J. R. Shearer J. Boyle K. Houmard J. A. (2009). Increased secretion and expression of myostatin in skeletal muscle from extremely obese women. Diabetes 58, 30–38. 10.2337/db08-0943 18835929 PMC2606890

[B41] Huang J. Romero-Suarez S. Lara N. Mo C. Kaja S. Brotto L. (2017). Crosstalk between MLO-Y4 osteocytes and C2C12 muscle cells is mediated by the Wnt/β-catenin pathway. JBMR Plus 1, 86–100. 10.1002/jbm4.10015 29104955 PMC5667655

[B42] Hu F. Lin Y. Zuo Y. Chen R. Luo S. Su Z. (2019). CCN1 induces adipogenic differentiation of fibro/adipogenic progenitors in a chronic kidney disease model. Biochem. Biophysical Res. Commun. 520, 385–391. 10.1016/j.bbrc.2019.10.047 31606201

[B43] Jackson W. M. Aragon A. B. Onodera J. Koehler S. M. Ji Y. Bulken-Hoover J. D. (2011). Cytokine expression in muscle following traumatic injury. J. Orthop. Res. 29, 1613–1620. 10.1002/jor.21354 21452302 PMC3150639

[B44] Jayasinghe S. A. L. Scheidt R. A. Sainburg R. L. (2022). Neural control of stopping and stabilizing the arm. Front. Integr. Neurosci. 16, 835852. 10.3389/fnint.2022.835852 35264934 PMC8899537

[B45] Jeffery E. C. Mann T. L. A. Pool J. A. Zhao Z. Morrison S. J. (2022). Bone marrow and periosteal skeletal stem/progenitor cells make distinct contributions to bone maintenance and repair. Cell Stem Cell 29, 1547–1561.e6. 10.1016/j.stem.2022.10.002 36272401

[B46] Jørgensen L. H. Petersson S. J. Sellathurai J. Andersen D. C. Thayssen S. Sant D. J. (2009). Secreted protein acidic and rich in cysteine (SPARC) in human skeletal muscle. J. Histochem Cytochem 57, 29–39. 10.1369/jhc.2008.951954 18796407 PMC2605719

[B47] Juban G. Saclier M. Yacoub-Youssef H. Kernou A. Arnold L. Boisson C. (2018). AMPK activation regulates LTBP4-dependent TGF-β1 secretion by pro-inflammatory macrophages and controls fibrosis in duchenne muscular dystrophy. Cell Rep. 25, 2163–2176. 10.1016/j.celrep.2018.10.077 30463013

[B48] Julien A. Kanagalingam A. Martínez-Sarrà E. Megret J. Luka M. Ménager M. (2021). Direct contribution of skeletal muscle mesenchymal progenitors to bone repair. Nat. Commun. 12, 2860. 10.1038/s41467-021-22842-5 34001878 PMC8128920

[B49] Jurdana M. (2021). Physical activity and cancer risk. Actual knowledge and possible biological mechanisms. Radiol. Oncol. 55, 7–17. 10.2478/raon-2020-0063 33885236 PMC7877262

[B50] Kamei Y. Hatazawa Y. Uchitomi R. Yoshimura R. Miura S. (2020). Regulation of skeletal muscle function by amino acids. Nutrients 12, 261. 10.3390/nu12010261 31963899 PMC7019684

[B51] Kellum E. Starr H. Arounleut P. Immel D. Fulzele S. Wenger K. (2009). Myostatin (GDF-8) deficiency increases fracture callus size, Sox-5 expression, and callus bone volume. Bone 44, 17–23. 10.1016/j.bone.2008.08.126 18852073 PMC2648293

[B52] Kessler E. Takahara K. Biniaminov L. Brusel M. Greenspan D. S. (1996). Bone morphogenetic protein-1: the type I procollagen C-proteinase. Science 271, 360–362. 10.1126/science.271.5247.360 8553073

[B53] Khan S. U. Ghafoor S. (2019). Myokines: discovery challenges and therapeutic impediments. J. Pak Med. Assoc. 69, 1014–1017. 31983736

[B54] Kim J. H. Kim D. Y. (2018). Aquarobic exercises improve the serum blood irisin and brain-derived neurotrophic factor levels in elderly women. Exp. Gerontol. 104, 60–65. 10.1016/j.exger.2018.01.024 29408452

[B55] Kim J. H. Sim J. H. Lee S. Seol M. A. Ye S. K. Shin H. M. (2017). Interleukin-7 induces osteoclast formation via STAT5, independent of receptor activator of NF-kappaB ligand. Front. Immunol. 8, 1376. 10.3389/fimmu.2017.01376 29104576 PMC5655015

[B56] Kim J. S. Galvão D. A. Newton R. U. Gray E. Taaffe D. R. (2021). Exercise-induced myokines and their effect on prostate cancer. Nat. Rev. Urol. 18, 519–542. 10.1038/s41585-021-00476-y 34158658

[B57] Kim S. J. Chang H. J. Volin M. V. Umar S. Van Raemdonck K. Chevalier A. (2020). Macrophages are the primary effector cells in IL-7-induced arthritis. Cell Mol. Immunol. 17, 728–740. 10.1038/s41423-019-0235-z 31197255 PMC7331600

[B58] Kim H. Wrann C. D. Jedrychowski M. Vidoni S. Kitase Y. Nagano K. (2018). Irisin mediates effects on bone and fat via αV integrin receptors. Cell 175, 1756–1768. 10.1016/j.cell.2018.10.025 30550785 PMC6298040

[B59] Kitase Y. Vallejo J. A. Gutheil W. Vemula H. Jähn K. Yi J. (2018). β-Aminoisobutyric acid, l-BAIBA, is a muscle-derived osteocyte survival factor. Cell Rep. 22, 1531–1544. 10.1016/j.celrep.2018.01.041 29425508 PMC5832359

[B60] Kjaer M. Secher N. H. Bangsbo J. Perko G. Horn A. Mohr T. (1996). Hormonal and metabolic responses to electrically induced cycling during epidural anesthesia in humans. J. Appl. Physiol. (1985) 80, 2156–2162. 10.1152/jappl.1996.80.6.2156 8806925

[B61] Kudo A. (2019). Periostin in bone biology. Adv. Exp. Med. Biol. 1132, 43–47. 10.1007/978-981-13-6657-4_5 31037623

[B62] Lee S. Y. An H. J. Kim J. M. Sung M. J. Kim D. K. Kim H. K. (2021). PINK1 deficiency impairs osteoblast differentiation through aberrant mitochondrial homeostasis. Stem Cell Res. Ther. 12, 589. 10.1186/s13287-021-02656-4 34823575 PMC8614054

[B63] Lees-Shepard J. B. Yamamoto M. Biswas A. A. Stoessel S. J. Nicholas S. E. Cogswell C. A. (2018). Activin-dependent signaling in fibro/adipogenic progenitors causes fibrodysplasia ossificans progressiva. Nat. Commun. 9, 471. 10.1038/s41467-018-02872-2 29396429 PMC5797136

[B64] Lemos D. R. Babaeijandaghi F. Low M. Chang C. K. Lee S. T. Fiore D. (2015). Nilotinib reduces muscle fibrosis in chronic muscle injury by promoting TNF-mediated apoptosis of fibro/adipogenic progenitors. Nat. Med. 21, 786–794. 10.1038/nm.3869 26053624

[B65] Lemos D. R. Duffield J. S. (2018). Tissue-resident mesenchymal stromal cells: implications for tissue-specific antifibrotic therapies. Sci. Transl. Med. 10, eaan5174. 10.1126/scitranslmed.aan5174 29386358

[B66] Leucht P. Kim J. B. Amasha R. James A. W. Girod S. Helms J. A. (2008). Embryonic origin and Hox status determine progenitor cell fate during adult bone regeneration. Development 135, 2845–2854. 10.1242/dev.023788 18653558

[B67] Lieu S. Hansen E. Dedini R. Behonick D. Werb Z. Miclau T. (2011). Impaired remodeling phase of fracture repair in the absence of matrix metalloproteinase-2. Dis. Model Mech. 4, 203–211. 10.1242/dmm.006304 21135056 PMC3046093

[B68] Liu Y. Lehar A. Rydzik R. Chandok H. Lee Y. S. Youngstrom D. W. (2021). Cell-type-specific neuromodulation guides synaptic credit assignment in a spiking neural network. Proc. Natl. Acad. Sci. U. S. A. 118, e2111821118. 10.1073/pnas.2111821118 34916291 PMC8713766

[B69] Li X. Jin L. Tan Y. (2021). Different roles of matrix metalloproteinase 2 in osteolysis of skeletal dysplasia and bone metastasis (Review). Mol. Med. Rep. 23, 70. 10.3892/mmr.2020.11708 33236155 PMC7716421

[B70] Loro E. Ramaswamy G. Chandra A. Tseng W. J. Mishra M. K. Shore E. M. (2017). IL15RA is required for osteoblast function and bone mineralization. Bone 103, 20–30. 10.1016/j.bone.2017.06.003 28602725 PMC5598756

[B71] Ludvig D. Whitmore M. W. Perreault E. J. (2022). Leveraging joint mechanics simplifies the neural control of movement. Front. Integr. Neurosci. 16, 802608. 10.3389/fnint.2022.802608 35387200 PMC8978895

[B72] Luo Z. Qi B. Sun Y. Chen Y. Lin J. Qin H. (2022). Engineering bioactive M2 macrophage-polarized, anti-inflammatory, miRNA-based liposomes for functional muscle repair: from exosomal mechanisms to biomaterials. Small 18, e2201957. 10.1002/smll.202201957 35802903

[B73] Maruyama M. Rhee C. Utsunomiya T. Zhang N. Ueno M. Yao Z. (2020). Modulation of the inflammatory response and bone healing. Front. Endocrinol. (Lausanne) 11, 386. 10.3389/fendo.2020.00386 32655495 PMC7325942

[B74] Matsushita Y. Nagata M. Kozloff K. M. Welch J. D. Mizuhashi K. Tokavanich N. (2020). A Wnt-mediated transformation of the bone marrow stromal cell identity orchestrates skeletal regeneration. Nat. Commun. 11, 332. 10.1038/s41467-019-14029-w 31949165 PMC6965122

[B75] Mazziotti G. Lania A. G. Canalis E. (2022). Skeletal disorders associated with the growth hormone-insulin-like growth factor 1 axis. Nat. Rev. Endocrinol. 18, 353–365. 10.1038/s41574-022-00649-8 35288658

[B76] Mcpherron A. C. Lawler A. M. Lee S. J. (1997). Regulation of skeletal muscle mass in mice by a new TGF-beta superfamily member. Nature 387, 83–90. 10.1038/387083a0 9139826

[B77] Mera P. Laue K. Ferron M. Confavreux C. Wei J. Galán-Díez M. (2016). Osteocalcin signaling in myofibers is necessary and sufficient for optimum adaptation to exercise. Cell Metab. 23, 1078–1092. 10.1016/j.cmet.2016.05.004 27304508 PMC4910629

[B78] Mizuhashi K. Ono W. Matsushita Y. Sakagami N. Takahashi A. Saunders T. L. (2018). Resting zone of the growth plate houses a unique class of skeletal stem cells. Nature 563, 254–258. 10.1038/s41586-018-0662-5 30401834 PMC6251707

[B79] Mo C. Zhao R. Vallejo J. Igwe O. Bonewald L. Wetmore L. (2015). Prostaglandin E2 promotes proliferation of skeletal muscle myoblasts via EP4 receptor activation. Cell Cycle 14, 1507–1516. 10.1080/15384101.2015.1026520 25785867 PMC4615122

[B80] Mohr T. Andersen J. L. Biering-Sørensen F. Galbo H. Bangsbo J. Wagner A. (1997). Long-term adaptation to electrically induced cycle training in severe spinal cord injured individuals. Spinal Cord. 35, 1–16. 10.1038/sj.sc.3100343 9025213

[B81] Mozzetta C. Consalvi S. Saccone V. Tierney M. Diamantini A. Mitchell K. J. (2013). Fibroadipogenic progenitors mediate the ability of HDAC inhibitors to promote regeneration in dystrophic muscles of young, but not old Mdx mice. EMBO Mol. Med. 5, 626–639. 10.1002/emmm.201202096 23505062 PMC3628105

[B82] Novais A. Chatzopoulou E. Chaussain C. Gorin C. (2021). The potential of FGF-2 in craniofacial bone tissue engineering: a review. Cells 10, 932. 10.3390/cells10040932 33920587 PMC8073160

[B83] Ogata Y. Kukita A. Kukita T. Komine M. Miyahara A. Miyazaki S. (1999). A novel role of IL-15 in the development of osteoclasts: inability to replace its activity with IL-2. J. Immunol. 162, 2754–2760. 10.4049/jimmunol.162.5.2754 10072521

[B84] Omosule C. L. Joseph D. Weiler B. Gremminger V. L. Silvey S. Jeong Y. (2022). Combinatorial inhibition of myostatin and activin A improves femoral bone properties in the G610C mouse model of osteogenesis imperfecta. J. Bone Min. Res. 37, 938–953. 10.1002/jbmr.4529 PMC10041862 35195284

[B85] Palermo A. Strollo R. Maddaloni E. Tuccinardi D. D'Onofrio L. Briganti S. I. (2015). Irisin is associated with osteoporotic fractures independently of bone mineral density, body composition or daily physical activity. Clin. Endocrinol. (Oxf) 82, 615–619. 10.1111/cen.12672 25400208

[B86] Pedersen B. K. Pedersen M. Krabbe K. S. Bruunsgaard H. Matthews V. B. Febbraio M. A. (2009). Role of exercise-induced brain-derived neurotrophic factor production in the regulation of energy homeostasis in mammals. Exp. Physiol. 94, 1153–1160. 10.1113/expphysiol.2009.048561 19748969

[B87] Phillips C. L. Jeong Y. (2018). Osteogenesis imperfecta: muscle-bone interactions when Bi-directionally compromised. Curr. Osteoporos. Rep. 16, 478–489. 10.1007/s11914-018-0456-6 29909596

[B88] Pratsinis H. Mavrogonatou E. Kletsas D. (2019). Scarless wound healing: from development to senescence. Adv. Drug Deliv. Rev. 146, 325–343. 10.1016/j.addr.2018.04.011 29654790

[B89] Qin H. Luo Z. Sun Y. He Z. Qi B. Chen Y. (2023). Low-intensity pulsed ultrasound promotes skeletal muscle regeneration via modulating the inflammatory immune microenvironment. Int. J. Biol. Sci. 19, 1123–1145. 10.7150/ijbs.79685 36923940 PMC10008697

[B90] Rauch F. Bailey D. A. Baxter-Jones A. Mirwald R. Faulkner R. (2004). The 'muscle-bone unit' during the pubertal growth spurt. Bone 34, 771–775. 10.1016/j.bone.2004.01.022 15121007

[B91] Rinkevich Y. Walmsley G. G. Hu M. S. Maan Z. N. Newman A. M. Drukker M. (2015). Skin fibrosis. Identification and isolation of a dermal lineage with intrinsic fibrogenic potential. Science 348, aaa2151. 10.1126/science.aaa2151 25883361 PMC5088503

[B92] Rodgers J. T. Schroeder M. D. Ma C. Rando T. A. (2017). HGFA is an injury-regulated systemic factor that induces the transition of stem cells into GAlert. Cell Rep. 19, 479–486. 10.1016/j.celrep.2017.03.066 28423312 PMC5468096

[B93] Sakai-Takemura F. Nogami K. Elhussieny A. Kawabata K. Maruyama Y. Hashimoto N. (2020). Prostaglandin EP2 receptor downstream of Notch signaling inhibits differentiation of human skeletal muscle progenitors in differentiation conditions. Commun. Biol. 3, 182. 10.1038/s42003-020-0904-6 32313117 PMC7171165

[B94] Sastourné-Arrey Q. Mathieu M. Contreras X. Monferran S. Bourlier V. Gil-Ortega M. (2023). Adipose tissue is a source of regenerative cells that augment the repair of skeletal muscle after injury. Nat. Commun. 14, 80. 10.1038/s41467-022-35524-7 36604419 PMC9816314

[B95] Sefton E. M. Kardon G. (2019). Connecting muscle development, birth defects, and evolution: an essential role for muscle connective tissue. Curr. Top. Dev. Biol. 132, 137–176. 10.1016/bs.ctdb.2018.12.004 30797508 PMC6449175

[B96] Serowoky M. A. Arata C. E. Crump J. G. Mariani F. V. (2020). Skeletal stem cells: insights into maintaining and regenerating the skeleton. Development 147, dev179325. 10.1242/dev.179325 32161063 PMC7075071

[B97] Severinsen M. C. K. Pedersen B. K. (2020). Muscle-organ crosstalk: the emerging roles of myokines. Endocr. Rev. 41, 594–609. 10.1210/endrev/bnaa016 32393961 PMC7288608

[B98] Shao X. Fu X. Yang J. Sui W. Li S. Yang W. (2023). The asymmetrical ESR1 signaling in muscle progenitor cells determines the progression of adolescent idiopathic scoliosis. Cell Discov. 9, 44. 10.1038/s41421-023-00531-5 37185898 PMC10130095

[B99] Shapiro S. A. Finnoff J. T. Awan T. M. Borg-Stein J. P. Harmon K. G. Herman D. C. (2022). Highlights from the American Medical Society for Sports Medicine position statement on responsible use of regenerative medicine and orthobiologics in sports medicine. Br. J. Sports Med. 56, 121–122. 10.1136/bjsports-2021-104887 34719428

[B100] Shapiro S. A. Master Z. Arthurs J. R. Mautner K. (2023). Tiered approach to considering orthobiologics for patients with musculoskeletal conditions. Br. J. Sports Med. 57, 179–180. 10.1136/bjsports-2022-106494 36650036 PMC9887351

[B101] Shore E. M. (2011). Osteoinductive signals and heterotopic ossification. J. Bone Min. Res. 26, 1163–1165. 10.1002/jbmr.404 21523827

[B102] Singhal V. Lawson E. A. Ackerman K. E. Fazeli P. K. Clarke H. Lee H. (2014). Irisin levels are lower in young amenorrheic athletes compared with eumenorrheic athletes and non-athletes and are associated with bone density and strength estimates. PLoS One 9, e100218. 10.1371/journal.pone.0100218 24926783 PMC4057451

[B103] Storlino G. Colaianni G. Sanesi L. Lippo L. Brunetti G. Errede M. (2020). Irisin prevents disuse-induced osteocyte apoptosis. J. Bone Min. Res. 35, 766–775. 10.1002/jbmr.3944 31826311

[B104] Suh J. Kim N. K. Lee S. H. Eom J. H. Lee Y. Park J. C. (2020). GDF11 promotes osteogenesis as opposed to MSTN, and follistatin, a MSTN/GDF11 inhibitor, increases muscle mass but weakens bone. Proc. Natl. Acad. Sci. U. S. A. 117, 4910–4920. 10.1073/pnas.1916034117 32071240 PMC7060712

[B105] Sun K. Jing X. Guo J. Yao X. Guo F. (2021). Mitophagy in degenerative joint diseases. Autophagy 17, 2082–2092. 10.1080/15548627.2020.1822097 32967533 PMC8496714

[B106] Sweeney H. L. Hammers D. W. (2018). Muscle contraction. Cold Spring Harb. Perspect. Biol. 10, a023200. 10.1101/cshperspect.a023200 29419405 PMC5793755

[B107] Sylvester A. D. Lautzenheiser S. G. Kramer P. A. (2021). A review of musculoskeletal modelling of human locomotion. Interface Focus 11, 20200060. 10.1098/rsfs.2020.0060 34938430 PMC8361578

[B108] Tanaka K. Matsumoto E. Higashimaki Y. Katagiri T. Sugimoto T. Seino S. (2012). Role of osteoglycin in the linkage between muscle and bone. J. Biol. Chem. 287, 11616–11628. 10.1074/jbc.M111.292193 22351757 PMC3320911

[B109] Tang L. Zhao T. Kang Y. An S. Fan X. Sun L. (2022). MSTN is an important myokine for weight-bearing training to attenuate bone loss in ovariectomized rats. J. Physiol. Biochem. 78, 61–72. 10.1007/s13105-021-00838-5 34453705

[B110] Udagawa N. Takahashi N. Katagiri T. Tamura T. Wada S. Findlay D. M. (1995). Interleukin (IL)-6 induction of osteoclast differentiation depends on IL-6 receptors expressed on osteoblastic cells but not on osteoclast progenitors. J. Exp. Med. 182, 1461–1468. 10.1084/jem.182.5.1461 7595216 PMC2192181

[B111] Veilleux L. N. Pouliot-Laforte A. Lemay M. Cheung M. S. Glorieux F. H. Rauch F. (2015). The functional muscle-bone unit in patients with osteogenesis imperfecta type I. Bone 79, 52–57. 10.1016/j.bone.2015.05.019 26004918

[B112] Vogt P. M. Boorboor P. Vaske B. Topsakal E. Schneider M. Muehlberger T. (2005). Significant angiogenic potential is present in the microenvironment of muscle flaps in humans. J. Reconstr. Microsurg 21, 517–523. 10.1055/s-2005-922429 16292726

[B113] Wang H. Zheng X. Zhang Y. Huang J. Zhou W. Li X. (2021a). The endocrine role of bone: novel functions of bone-derived cytokines. Biochem. Pharmacol. 183, 114308. 10.1016/j.bcp.2020.114308 33137323

[B114] Wang L. You X. Lotinun S. Zhang L. Wu N. Zou W. (2020). Mechanical sensing protein PIEZO1 regulates bone homeostasis via osteoblast-osteoclast crosstalk. Nat. Commun. 11, 282. 10.1038/s41467-019-14146-6 31941964 PMC6962448

[B115] Wang T. Wagner A. Gehwolf R. Yan W. Passini F. S. Thien C. (2021b). Load-induced regulation of tendon homeostasis by SPARC, a genetic predisposition factor for tendon and ligament injuries. Sci. Transl. Med. 13, eabe5738. 10.1126/scitranslmed.abe5738 33627488

[B116] Waning D. L. Mohammad K. S. Reiken S. Xie W. Andersson D. C. John S. (2015). Excess TGF-β mediates muscle weakness associated with bone metastases in mice. Nat. Med. 21, 1262–1271. 10.1038/nm.3961 26457758 PMC4636436

[B117] Waseem R. Shamsi A. Mohammad T. Hassan M. I. Kazim S. N. Chaudhary A. A. (2022). FNDC5/Irisin: physiology and pathophysiology. Molecules 27, 1118. 10.3390/molecules27031118 35164383 PMC8838669

[B118] Wu G. Zhang X. Gao F. (2021). The epigenetic landscape of exercise in cardiac health and disease. J. Sport Health Sci. 10, 648–659. 10.1016/j.jshs.2020.12.003 33333247 PMC8724625

[B119] Xiong J. Onal M. Jilka R. L. Weinstein R. S. Manolagas S. C. O'Brien C. A. (2011). Matrix-embedded cells control osteoclast formation. Nat. Med. 17, 1235–1241. 10.1038/nm.2448 21909103 PMC3192296

[B120] Zhang J. Valverde P. Zhu X. Murray D. Wu Y. Yu L. (2017a). Exercise-induced irisin in bone and systemic irisin administration reveal new regulatory mechanisms of bone metabolism. Bone Res. 5, 16056. 10.1038/boneres.2016.56 28944087 PMC5605767

[B121] Zhang Z. Hu P. Wang Z. Qiu X. Chen Y. (2020). BDNF promoted osteoblast migration and fracture healing by up-regulating integrin β1 via TrkB-mediated ERK1/2 and AKT signalling. J. Cell Mol. Med. 24, 10792–10802. 10.1111/jcmm.15704 32803867 PMC7521296

[B122] Zhang Z. Zhang Y. Zhou Z. Shi H. Qiu X. Xiong J. (2017b). BDNF regulates the expression and secretion of VEGF from osteoblasts via the TrkB/ERK1/2 signaling pathway during fracture healing. Mol. Med. Rep. 15, 1362–1367. 10.3892/mmr.2017.6110 28098876

[B123] Zhao H. Feng J. Ho T. V. Grimes W. Urata M. Chai Y. (2015). The suture provides a niche for mesenchymal stem cells of craniofacial bones. Nat. Cell Biol. 17, 386–396. 10.1038/ncb3139 25799059 PMC4380556

[B124] Zhi X. Chen Q. Song S. Gu Z. Wei W. Chen H. (2020). Myostatin promotes osteoclastogenesis by regulating Ccdc50 gene expression and RANKL-induced NF-κB and MAPK pathways. Front. Pharmacol. 11, 565163. 10.3389/fphar.2020.565163 33536903 PMC7849192

[B125] Zhou B. O. Yue R. Murphy M. M. Peyer J. G. Morrison S. J. (2014). Leptin-receptor-expressing mesenchymal stromal cells represent the main source of bone formed by adult bone marrow. Cell Stem Cell 15, 154–168. 10.1016/j.stem.2014.06.008 24953181 PMC4127103

[B126] Zhou S. Qian B. Wang L. Zhang C. Hogan M. V. Li H. (2018). Altered bone-regulating myokine expression in skeletal muscle of Duchenne muscular dystrophy mouse models. Muscle Nerve 58, 573–582. 10.1002/mus.26195 30028902

