# Supplementary material for: The reciprocity of skeletal muscle and bone: an evolving view from mechanical coupling, secretory crosstalk to stem cell exchange
Source: Front Physiol. 2024 Mar 4;15:1349253. doi: 10.3389/fphys.2024.1349253 (PMC10949226; doi:10.3389/fphys.2024.1349253)
Supplement: Supplementary file 1 [file Table1.docx]

**The reciprocity of skeletal muscle and bone: an evolving view from mechanical coupling, secretory crosstalk to stem cell exchange**

Hao Sui, Jinfeng Dou, Bing Shi, Xu Cheng*

**Supplementary Table 1. Myokines and osteokines facillitating muscle-bone secretory interaction.**

| Category | | | Secretory cell | Description | Reference |
| --- | --- | --- | --- | --- | --- |
| Myokines | Bone formation myokines | IGF-1 | Myofibers | Enhances the differentiated function of osteoblasts and prevents cell apoptosis | (Mazziotti et al., 2022, Chen et al., 2022) |
|  |  | FGF-2 | Myofibers | Promotes proliferation and differentiation of osteoprogenitor cells | ( Chen et al., 2022, Novais et al., 2021) |
|  |  | Irisin | Myofibers | Induces osteoblastogenesis;  Prevents osteocyte apoptosis;  Promotes bone resorption by increasing differentiation of osteoclast | (Zhang et al., 2017a)  (Storlino et al., 2020, Kim et al., 2018)  (Estell et al., 2020) |
|  |  | SPARC | Myofibers | Promotes fibrocartilage mineralization | (Zhou et al., 2018) |
|  |  | MMP-2 | Myofibers | Affects the later stages of fracture healing tissue remodeling | (Lieu et al., 2011) |
|  |  | BMP-1 | Myofibers | May be involved in ectopic ossification | (Jackson et al., 2011) |
|  |  | BDNF | Myofibers | Stimulates osteoblasts migration and VEGF expression to promote fracture healing | (Zhang et al., 2017b) |
|  |  | BAIBA | Myofibers | Prevents cell death of osteocyte | (Zhang et al., 2017b) |
|  |  | Osteoglycin | Myofibers | Promotes bone formation | (Tanaka et al., 2012) |
|  | bone resorption myokines | Myostatin | Myofibers | Impedes osteoblast differentiation and activates osteoclast maturation | (Zhi et al., 2020, Tang et al., 2022, Suh et al., 2020, Omosule et al., 2022, Liu et al., 2021, Bialek et al., 2014) |
|  |  | IL-6 | Myofibers | Drives osteoclastogenesis in IL-6 receptor- expressing cells | (Chowdhury et al., 2020, Udagawa et al., 1995) |
|  |  | IL-7 | Unknown | Induces osteoclastogenesis and promote bone resorption | (Kim et al., 2017, Kim et al., 2020) |
|  |  | IL-15 | Unknown | Promotes osteoclastogensis in the early differentiation process;  Licenses circulating NK cells to eliminate mature osteoclasts and maintains osteoblast mineralization capacity | (Ogata et al., 1999)  (Feng et al., 2015, Loro et al., 2017) |
| Osteokines | Muscle growth osteokines | PGE-2 | Osteocytes | Enhances myoblast proliferation | (Mo et al., 2015) |
|  |  | Osteocalcin | Osteoblasts and osteocytes | Positive impact on muscle mass and function | (Mera et al., 2016) |
|  |  | Wnt3a | Osteocytes | Induces myogenesis | (Huang et al., 2017) |
|  | Muscle degeneration osteokines | TGFβ | Osteoblasts | Excessive release of TGFβ from bones causes muscle weakness | (Waning et al., 2015) |
|  |  | RANKL | / | RANKL inhibitor increase muscle mass and function. | (Huang et al., 2017, Dufresne et al., 2018) |
